# Cervical Screening Reminders for Aboriginal and Torres Strait Islander Women in Primary Care—Randomised Controlled Trial of Letter vs. Phone/SMS Reminders

**DOI:** 10.3390/ijerph20136257

**Published:** 2023-06-29

**Authors:** Rowena Ivers, Trish Levett, Kyla Wynn

**Affiliations:** 1 Illawarra Aboriginal Medical Service, 150 Church St, Wollongong, NSW 2500, Australia; 2 Graduate School of Medicine, University of Wollongong, Wollongong, NSW 2522, Australia

**Keywords:** cervical screening, Aboriginal, Torres Strait Islander, reminder, primary care, health promotion

## Abstract

(1) Background: Aboriginal women have a higher mortality from cervical cancer, yet cervical screening rates are lower than for other Australian women. (2) Methods: A randomised controlled trial of reminder letter vs. phone call/SMS for routine cervical screening testing in an Aboriginal Community Controlled Health Organisation in NSW. (3) Results: 256 women aged between 25 and 74 who were due for cervical screening were randomised to receive a reminder letter (and up to two further letters for non-responders) or a phone call (followed by up to two SMS) to attend the screening. A total of 15 women (12.5%) attended for cervical screening test within 3 months following a letter, and 24 women (17.6%) after a phone call/SMS reminder; this difference was not significant (*p* = 0.252). Time spent on sending letters vs. phone calls/SMS was similar; the cost was lowest for SMS. (4) Conclusion: Response to reminders was lower than expected. While there was no significant difference in effectiveness in letter vs. phone call/SMS for cervical screening recalls, reminder systems, including opportunistic reminders, can play a role in encouraging women to participate in screening programs in conjunction with national screening registers. The choice of reminder type should be left to service and consumer preference.

## 1. Introduction

Cervical cancer screening, along with human papillomavirus (HPV) vaccination, can play a role in the elimination of cervical cancer. Although the age-standardised incidence of cervical cancer was 2.2 times higher for Aboriginal and Torres Strait Islander women than for other Australian women (13.9 per 100,000 vs. 6.4 per 100,100 in 2011-5) [1] and mortality from cervical cancer was higher than other Australian women [2], there are opportunities to improve outcomes, with Aboriginal Community Controlled Health Organisations (ACCHOs) playing a role.

The National Cervical Screening Program aims to prevent cervical cancer by early detection through screening. Screening is available to women and people with a cervix aged between 25 and 74 who have ever had sexual contact [3]. Accurate estimation of screening participation for Aboriginal and Torres Strait Islander women is not possible, as Aboriginal or Torres Strait Islander status is not routinely recorded on the pathology forms from which cervical screening register data is collated [4].

Where data was collected, the cervical screening participation rate for Aboriginal and Torres Strait Islander women is 40%, but participation rates range from 19.9% to 63.5% [5,6,7]. By comparison, in 2016-7, the estimated cervical screening participation rate for Australian women aged 20–69 was 54–56% [8].

The National Cancer Screening Register (NCSR) (incorporating previously state-based cervical screening registers) provides reminders to participants regarding screening. In 2016, of more than one million women who were sent a reminder letter by a cervical screening register, 31% were rescreened within 3 months [8]. 

Primary care, including ACCHOs, plays an important role in preventive care, including in promoting screening for cervical cancer; in a study of 1,107,233 women in Queensland in 2013-7, ACCHOs recorded higher screening rates than mainstream services [9].

Reminder systems have been used in Aboriginal health services for over thirty years [10,11,12] and can complement reminders from screening registers. One ACCHO in the Northern Territory assessed the effect of phone, mailed letters, or opportunistic reminders, concluding that response rates were low and that opportunistic reminders were also critical in promoting screening [11]. This service utilised medical record tagging, that is, noting in the medical record that a person was due for cervical screening, so that if they attended for another reason, a health professional could offer them screening. 

Under-screened and never-screened women are at higher risk of cervical cancer. Some women decline a cervical screening test (CST) collected by speculum because of its invasive nature. In 2017, self-collected CST became available for women over 30 years of age who were 2 years overdue for screening and who had declined a speculum examination. In a qualitative study involving interviews with 94 Indigenous women aged 25–74 in NSW, many women were not aware of the new option for self-collected specimens, and women felt unwilling to complete a self-collected test for fear of using an incorrect method [13]. By contrast, another study of 215 women in one remote Aboriginal community showed that a nurse-led program to support self-collected CST was successful and well-accepted by women [14]. Reminder systems thus need to promote this option to eligible women and provide education and reassurance about this mode of collection.

Primary health services also play a role in reviewing those with symptoms of cervical cancer, for example, irregular vaginal bleeding or pain, by providing investigation and referral to specialist gynaecological services, for example, for colposcopy. 

In contrast to reminders, which are used for preventive care, recall services are designed to inform clients of abnormal results, and to encourage them to attend for follow-up treatment. Primary health services provide recalls for abnormal results on cervical screening tests or other investigations, for example, by phoning at different times of the day, mailing a recall letter, and if the person is not contactable, mailing a registered mail letter, to ensure that at least three documented attempts have been made at contacting the person.

Reminders for preventive care are considered part of quality primary care but may be both time-consuming and are not renumerated by funding systems. The aim of this project was to determine which mode of reminder was most likely to result in presentation for cervical screening and was most cost-effective as part of quality improvement. 

## 2. Materials and Methods

This project consisted of a small, randomised trial of reminder letters vs. phone calls/SMS for routine cervical screening testing in an ACCHO in NSW.

Eligible clients included women aged 25–74, who were regular clients at the ACCHO who had presented for any condition in the previous three years, and who were eligible for cervical cancer screening. 

**Figure 1:**
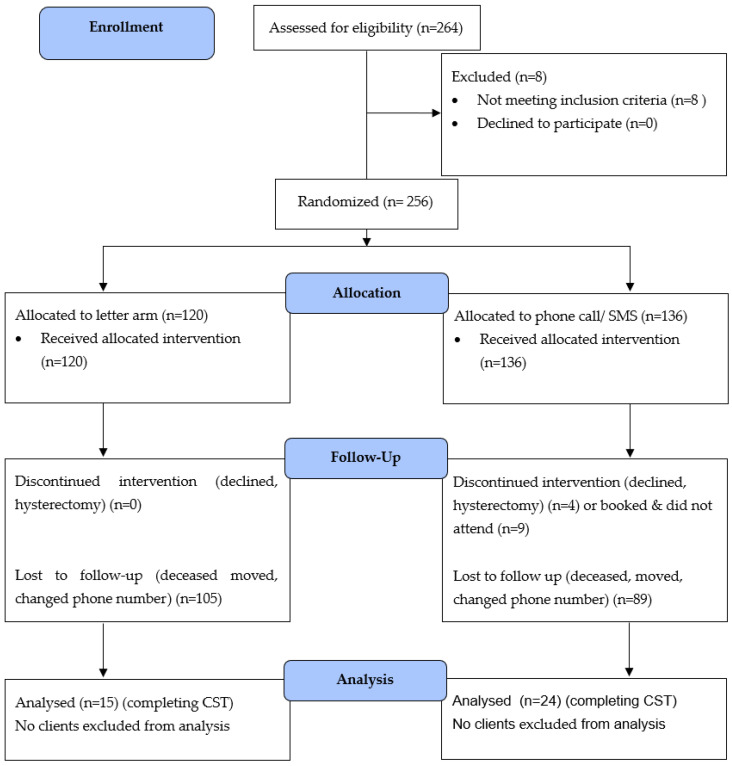
CONSORT diagram: letter vs. phone call/SMS reminder.

Exclusions included women who had had recent abnormal screening results and were referred for gynaecological follow-up, for example, for colposcopy. Women who had had a hysterectomy and no longer required cervical screening were excluded. (Refer to Figure 1).

Using the electronic medical record software, Communicare [15], the Cancer Care Team (Aboriginal Health Worker (AHW), cancer care counsellor/enrolled nurse, and general practitioner) conducted monthly reporting on those eligible for screening as part of routine quality improvement. The team first reviewed reminders for all apparently eligible participants, including those who had no reminder entered, and who were regular clients of the service. Eligibility was then reviewed against the state cervical screening register to ensure clients had not been screened elsewhere. Communicare was then used to generate a ‘report’ for those due for cervical screening.

For the purpose of the trial, data was entered manually into an Excel spreadsheet, and stored in a password-locked, secure server with back-up. Participants were given a study identification number, which was stored in a separate secure file. The sample size was not calculated prior to commencement as this study was limited by the available sample size within this service. 

Randomisation was undertaken using an online randomising program, and participants were randomised to each arm in a 1:1 ratio. One arm received a reminder letter, followed by two further reminder letters unless an appointment was booked, and the other arm received a phone call, then two SMS, unless an appointment was booked. 

Women who were eligible for self-collect cervical cancer screening (women over 30, who were at least two years overdue for screening, who had declined speculum collection, and who had no recent abnormal tests) were mailed or verbally given additional information about options for self-collection. At the time of the trial, self-collection was only available for this subset of women.

The cervical screening was delivered by female staff, including AHWs and Aboriginal nurses, with emphasis on making care accessible, for example, by providing free transport. Incentives were offered to women for participating in ‘Women’s Business’ or other preventive care, such as the annual preventive health assessment available to Aboriginal or Torres Strait Islander participants, for example, gifts or T-shirts. 

As part of routine care, women with abnormal results were followed as per national guidelines, for example, to discuss the results and then refer for colposcopy. Abnormal results were delivered via the recall system; that is, to provide at least three attempts at contacting those with abnormal results, all documented in the electronic medical record.

Analysis was undertaken, first with descriptive statistics (demographics, previous number of reminders, previous abnormalities), then using SPSS for calculation of correlation of reminder type versus presentation for cervical screening. The outcome for measurement included completion of cervical screening within three months of the first reminder. Secondary outcomes included the presence of HPV or cervical abnormalities.

Consumer feedback was gathered informally through program feedback forms. 

Ethics approval was given by the AHMRC ethics committee (1420/18), following support of the project by the ACCHO. A waiver of consent was allowed, as these activities constituted a part of routine service delivery, and both modes of reminder were already being used. The trial was registered (ACTRN12618001652268). 

## 3. Results

Two hundred and fifty-six current clients aged between 25 and 74 who were due for cervical screening were included. Eight women were excluded due to previous hysterectomies or current referrals.

The age range of participants was 25–74 years, with a median age of 43 years (letter arm 44 years, telephone/SMS arm 40.5 years). All but two women identified as Aboriginal and/or Torres Strait Islander, with those who were not Aboriginal or Torres Strait Islander both in the letter arm. Non-Aboriginal family members of Aboriginal clients were routinely included in service data collection. 

Clients had previously received between 0 and 12 reminders for cervical screening (via various methods, including letters, phone calls, SMS, or opportunistic reminder). Of the participants, 120 were randomised by letter and 136 to phone call/SMS. 

Fifteen women (12.5%) attended CST within 3 months following a letter, and 24 women (17.6%) after a phone call/SMS reminder; while numerically more women responded to SMS, this difference was not significant (*p* = 0.252), probably due to the small sample size. Fourteen women had not had a previous CST, or not had one within the previous 4 years. 

Eight women in the letter arm responded after the first reminder, and others took further reminders to present. Only one woman in the letter arm booked and did not attend an appointment. Twenty women in the telephone/SMS groups responded after the first reminder and completed CST, with a further 10 booking in but not attending. All of those who did not attend were given additional reminders to book.

There were a range of reasons for not attending screening noted by clinic staff. One woman had had a cervical cancer screening test performed elsewhere in the period between contact with the Register and a reminder letter being received. One woman with intellectual disability and blindness had no telephone, and no street address. One woman had a lengthy intensive care unit admission during the study period. One had a hysterectomy for menorrhagia during the study period, and another woman was referred to a gynaecologist for symptoms suggestive of endometrial cancer. Two had moved to residential aged care and had new medical providers. Four others had moved, and street addresses or phone numbers were incorrect. An additional four women were homeless and had no street address or phone. Staff noted that many women who did not attend had serious mental illness or a history of trauma. Two women were not contactable as they were incarcerated. One person, unbeknownst to the team, was deceased at the commencement of the study, and another woman passed away during the study.

Three clients actively declined CST, including one client (letter arm) who had HSIL in the past (with clear CST since HSIL diagnosis) who actively declined CST follow-up, despite lengthy counselling. Reminders continued after the intervention period as part of routine clinical activities. Two women in the phone call/SMS arm actively declined CST. 

### 3.1. Cervical Screening Results

Two women had had previous cervical cancer, 5 HSIL, 3 LSIL, and two had had previously had HPV previously reported on CSTs. 

One woman with a past history of cervical cancer and hysterectomy returned an intermediate-risk result, which was expected as she was scheduled for an annual review. One woman returned a test showing high-risk HPV, entered the recall system for abnormal results, and was referred for colposcopy. One woman’s CST was reported as being unsatisfactory and was transferred to the recall system for follow-up for repeat CST. The results for two women were unknown as CST was completed elsewhere.

The team considered that some reminders might have been delivered to women who had never been sexually active, who then disclosed this when they presented for care and who thus did not require a CST. Information regarding eligibility would normally be recorded manually in a discrete manner but did not appear to apply to women included in this sample.

The authors were not aware that this study included people with a cervix who did not identify as female, although the electronic medical record software used was not able to accurately capture these data. 

### 3.2. Consumer Feedback

Participants commented favourably on their experience during Women’s health month, although none commented specifically on the nature of the reminder method.

### 3.3. Cost

The time spent on letters vs. SMS by experienced staff was similar (mean 34 vs. 32 s for median time for five of each mode), although women were more likely to respond and book for CST following phone calls. The cost of letter printing and mailing (AUD1.17) was more than the cost of a phone call or SMS (AUD0.17). 

## 4. Discussion

Reminders for cervical screening were conducted as part of routine care; the randomised design was conducted as part of quality improvement to consider the best outcomes and use of staff time in conducting the program.

Limitations of this study included the small sample size and that it was delivered in a single site. 

The first step of this project included reviewing the electronic medical record of all regular clients and reviewing eligibility for CST against the state register (now incorporated into the NCSR). Some people may not access primary care regularly and thus may not be included in regular prevention programs within health services and thus not have reminders for cervical screening recorded in their electronic medical record. Those who have never undertaken cervical screening or who have declined enrolment may not be listed on the state or national cervical screening registers.

At the time of this study, contacting the state cervical screening register to check eligibility could be conducted for individuals by telephone (not feasible for large samples of women) or by faxed/mailed request (feasible but longer response time). Updates in electronic medical record software may mean that linkage with the NCSR is possible, allowing instant review of eligibility, for example, at the time of an annual preventive Health Assessment. 

The study then involved sending reminders to those who were due for screening. The response to reminders was lower than expected (17.6% and 12.5%) after up to three reminders, compared to 31% for other Australian women [8]. Hunt’s study, also based on an ACCHO, showed low response levels, with only 2% of participants responding to reminder letters [11]. 

Several barriers were noted to letter reminders, including slow delivery times (anecdotal reports of taking two weeks for letters to be delivered) and a change of address. Telephone reminders allowed instant booking of appointments and transport. The team noted that phone calls from unidentified numbers might be ignored and purposely called from numbers that were known to be that of the health service. The team noted that SMS was likely to be responded to as workers could identify themselves. Women were more likely to book an appointment with a phone call/SMS, but a number of women then did not attend, possibly because they felt that they felt pressured to book in.

Some women were not contactable due to a lack of address or phone number recorded in the electronic medical record; reception staff in health services play an important role in reviewing and updating contact details at every visit. Mobility was high among clients, highlighting the need for a formal transfer of medical records, for example, to new practices, aged care providers, and correctional settings. 

Women who did not respond to three reminders were not likely to respond to further reminder attempts, and reminders beyond this point appeared to be futile. While reminders are not legally required, quality primary care services are able to promote prevention activities by providing reminders, and these supplement reminders sent by the NCSR. 

As per Hunt’s study [11], opportunistic reminders and ‘file tagging,’ that is, ensuring reminders are recorded in the electronic medical record, are also likely to play a role in promoting screening. Verbal reminders were routinely given at this service when clients presented for care, and were recorded in the electronic medical record. Services could consider a combination of reminder types (for example, to ensure that women without an address or without a telephone receive a reminder), or record consumer preference for reminder type. 

This cervical screening program was delivered in the context of an ACCHO. This service used to care that was considered culturally appropriate, including calls from Aboriginal workers, provided transport from female drivers, and conducted promotional activities, such as providing culturally appropriate health promotion material and incentives to make women’s health services respectful and welcoming. All services were free of charge. McLachlan et al. interviewed women and found that clear explanations from health providers about the process of testing, trusting relationships with health providers, and recognition of participants’ past experiences, including traumatic experiences, had the potential to increase screening [16].

This reminder program was initiated in the context of an integrated cancer care team; that is, the coordination of screening programs, support for clients through diagnosis (for example, recall to discuss abnormal results), treatment (for example, assisting in arranging appointments and accompanying vulnerable clients for colposcopy) and during cancer survivorship or even to end of life (although no participants were diagnosed with, or died from cervical cancer during the course of this study). This meant that women who received an abnormal result on cervical screening could be supported through referral to colposcopy, surgery, or other treatment, by the same team, ensuring continuity of care and that the woman was supported by trusted team members.

This study promoted self-collected tests to women who were eligible at the time, which is under-screened women who had declined sample collection with a speculum. Self-collected CST is now offered to all people with a cervix aged 25–74 in Australia, and this may mean cervical screening is acceptable to more women, especially those who have declined a speculum examination. Self-collection may be more amenable to group screening, for example, ‘well-women days’ led by nurses or AHWs, supported by general practitioners. Outreach services, for example, delivered to a person’s home, can make screening more accessible [14].

As for previous studies, this trial demonstrated that some women experienced additional barriers to participation in preventive care. The qualitative data presented here concurs with qualitative data collected elsewhere [16]; women with mental health conditions, and intellectual disability, who were homeless or who were incarcerated faced barriers to participation and may require additional support. Speculum examination may have been a barrier to screening for women who had experienced previous sexual assault, although data on this was not collected as part of this study. 

Reminder systems also need to consider women who are not eligible for CST, for example, those who have never been sexually active, and consider respectful ways to remove them from reminder systems. Registration in medical records should ideally allow the nomination of a range of gender identification but be able to identify if clients have a cervix and require screening.

Few abnormal findings were identified on CSTs collected in this small sample. In another study that analysed data for 23,482 female Queensland residents aged 20–69 years who had one or more Pap smears during 2000–2011, the prevalence of histologically confirmed high-grade abnormalities for Aboriginal Torres Strait Islander women was 16.6 per 1000 women screened, 95% confidence interval [CI] 14.6–18.9 [7]. During the implementation of self-collected CST in a remote community [14], 18.1% of women had a positive result, reflecting that under or never–screened women may show a higher prevalence of abnormalities. 

### 4.1. Cost

SMS appeared to have the lowest cost and to require less time than other reminder types. At the time of this study, automated SMS reminders were not possible on the version of Communicare used; however, they were available in other commercial software, and this was likely to be even more cost and time efficient. 

### 4.2. Quality Improvement

This study was conducted as part of quality improvement activities. As of August 2019, cervical screening was one of the core items collected from consenting medical practices by Primary Health Networks as part of the Practice Incentive Programs Quality Improvement Incentive [17]. Cervical screening can be a target program for continual quality improvement, including in ACCHOs [18]. The availability of universal access to self-collected cervical screening may present opportunities for quality improvement targeted at never or under-screened women. 

Other public health measures also contribute to lowering the incidence of cervical cancer, including routine HPV vaccination. HPV vaccine is free under the National Immunisation Program through school-based programs for children aged 12–13, with ACCHOs providing access to HPV vaccination for adolescents who had not received vaccination in school programs. There is already evidence that HPV vaccination has reduced the incidence of HPV infection, a precursor of cervical cancer, among Australian Aboriginal and Torres Strait Islander women. Elimination or massive reduction in cervical cancer prevalence may be possible through a combination of HPV vaccination programs, improved delivery of screening programs, as well as improved treatment [19]. 

## 5. Conclusions

While there was no significant difference in effectiveness in letter vs. phone call/SMS for cervical screening reminders, phone calls/SMS were numerically superior in this small sample. Reminder systems can play a role in encouraging women to participate in screening programs in conjunction with national screening registers and opportunistic reminders regarding cervical screening when presenting for care. The choice of reminder type should be left to service preference and could consider consumer preference. Reminders for cervical screening, and delivery of cervical screening programs, together with human papillomavirus vaccination programs, and new treatments for cervical cancer, can together assist progress towards elimination of cervical cancer for Aboriginal and Torres Strait Islander people.

## Data Availability

The data presented in this study are available on request from the corresponding author. The data are not publicly available as they are part of quality improvement data retained in medical services.
